# The Philosophy of the Moving Powers of the Blood

**Published:** 1845-04

**Authors:** 


### The Philosophy of the Moving Powers of the Blood. By *G. Calvert Holland*, M.D., Physician Extraordinary to the Sheffield General Infirmary; formerly President of the Hunterian and Royal Physical Societies, Edinburgh, &c. &c. 8vo. pp. 308. London, Churchill, 1844.

The title of this work is imposing, and it is probably owing to our expectations having been proportionally raised, that we have experienced some degree of disappointment in perusing it. Dr. Holland, in his preface, remarks that "to enforce truths a-head of the seeming necessities of the age, is to receive either the contempt or the persecution of mankind."

That men should closely scrutinize the evidence and reasoning by the force of which it is wished they should abandon opinions founded not upon mere authority, but upon investigations marked by talent and conducted with care, is not only natural but desirable: but that there is in the times in which we live any other disinclination beyond that stated to the reception of novel opinions and doctrines, we must take leave to doubt. However this may be, we can answer for ourselves that we have no special reverence for things because they are old, nor for names because they are high; and therefore we were quite prepared to abandon Harvey, Haller, Magendie, Müller, M. Hall, and the many other men of mark, who are arrayed together in Dr. Holland's table of "Contents" as propagators of error and fallacy, but upon one condition, that the author should afford us better reasons for our new belief than for our former faith, and to this limited extent we trust we may be permitted to retain our orthodoxy.

In the work before us, most of the experiments that have been performed of late years, relating to the powers which accomplish the circulation of the blood, are criticised with much minuteness; and although we feel ourselves called upon to dissent from many of the author's conclusions, it is right to state that several of the objections urged evince much penetration, and are well worthy of consideration.

A leading object with Dr. Holland is to show the insufficiency of the celebrated experiments of Magendie, by which that eminent physiologist endeavoured to prove that the power of the left ventricle of the heart operated not only in the propulsion of the arterial, but likewise of the venous blood. The principal of these experiments, as most of our readers are aware, was as follows: a ligature was applied round the thigh of a dog, excluding only the femoral artery and vein; the vein was then separately tied near the groin, and a slight puncture was made into this vessel; a considerable jet of blood flowed out, but upon pressing the artery between the fingers, although the jet continued for some instants, it soon stopped, notwithstanding the whole length of the vein was full; upon removing the pressure the jet was re-established, as soon as the blood, injected by the heart, had arrived at the last divisions of the arteries. Upon this experiment the author observes:—

"The cessation of the jet is no evidence that it arises from interruption to the influence of the heart. The same result would occur were the capillaries the cause of venous circulation. The stream flows as long as the capillaries receive blood. If none exist between the roots of the vein and the compressed artery, whence is the power to be derived to act on the contents of the vein?

"In the attempt to prove that the venous current is propelled by the heart alone, in common justice to the capillaries they ought not to be deprived of blood. It is unphilosophical to withhold this, by which they are stimulated to contraction, and then to accuse them of the want of power. Were the physiologist to prevent the blood flowing into the left auricle, and observing the arteries full to distension, to call attention to the fact, as evidence that the contents of these vessels were not at all influenced by the heart, the inference would naturally be objected to. But the capillaries are placed precisely in this situation. There is no difference between them and the heart in the case supposed. The artery, from which they receive their stimulus and source of contraction, is empty, and, consequently, the supply is cut off. There is a column of blood in advance of them, but there is none, *a-tergo*, by which to act upon it." P. 171.

These objections to us are not satisfactory, for it cannot fairly be inferred from the account of the experiment that, although the main artery was seen to contract and thus to become empty, the vast plexus of small arteries and capillaries of the limb was also in a state of vacuity, and consequently that the latter vessels wanted the necessary condition of their assumed action, namely, the presence of blood. On the contrary, this and other similar experiments are more reasonably explained upon the assumption, that the vis-à-tergo afforded by the left ventricle is necessary to the venous circulation. The well-known fact, that if, in venesection, the arm is bound up so tightly as to compress the brachial artery, after the first jet no blood will flow, although it is certain the limb is full of blood, is a strong corroboration of the truth of M. Magendie's deduction.

But there are others, and more decided proofs, that the heart's propulsive force can influence the veins. One of the most satisfactory of these is the occasional occurrence of a venous pulse, caused, not like the more ordinary one by a reflux of blood from the right auricle, but by a *vis-à-tergo*. M. Martin Solon has recently communicated some very instructive cases, (Bulletin de l'Acad. Roy. de Med. 1844, p. 102,) from which we extract the following particulars. One of these cases occurred in a young man, aged 23, the subject of pleuro-pneumonia on both sides: the pulse of the radial artery 110, full and strong; the impulse of the heart strong and energetic; the dorsal veins of the hands, which were prominent, rounded and of a slightly blueish and rose colour, presented a movement of diastole and systole sensible to the sight and appreciable by the touch: these pulsatile motions were isochronous with the radial pulse; they ceased, and the veins diminished in volume, when pressure was made upon these vessels towards the fingers, whilst, on the contrary, the motions persisted when pressure was applied to the wrist; compression of the brachial artery also caused the pulsations to cease. Other and similar cases are related, and those of Dr. Ward (Medical Gazette, 1832, p. 376) and of Dr. Graves are quoted.

Dr. Ward, in the paper referred to, attributed this interesting phenomenon "to the excessive action of the heart, pushing the thin and impoverished blood through the capillaries straight on into the veins;" a view which is adopted by M. Solon, though we are sorry to say, without any reference to the earlier English observer. There can be no doubt that the causes assigned by Dr. Ward must be operative; but the obstruction à fronte to the free return of the venous blood, dependent upon the embarrassment of the pulmonary circulation, which we believe always exists in these cases, doubtless is an additional and a necessary condition.

But whatever may be the cause of this form of venous pulse, the latter is an unquestionable proof that the power of the heart can, under certain circumstances, influence the circulation in the veins.

Dr. Holland also calls in question the opinion of physiologists, resting upon the experiments of Poiseuille and other evidence, that the elasticity of the arteries is the main cause by which the periodic impulse of the heart is converted into a permanent force, and by which the current of the blood acquires the uniformity observed in the capillary vessels. The author remarks, "this experiment (Poiseuille's) proves that an artery, when greatly distended, as in this instance, is capable of re-acting on its contents; but no conclusion can be drawn from the fact in reference to the agency of elasticity in the circulation of the blood." In this, as on most other points, we do not agree with the writer, and for this, among other reasons, because we do not think it probable that such a high degree of elasticity would have been conferred upon the arterial tunics, so great indeed as to have become proverbial, if it were not designed to perform some important office in the mechanism of the circulation.

Dr. Holland, in criticising the researches of Dr. M. Hall and others, objects with justice to conclusions being drawn concerning the nature of the capillary circulation, after great disturbance has been induced by the experiments themselves. If, for instance, a ligature be applied around the leg of a frog, what satisfactory results can be obtained when, on the one hand, the supply of blood through the arteries is cut off, and, on the other, the exit through the veins is totally obstructed? Herein, in fact, is comprised the essence of the author's charges against experimentalists: "the analysis has constantly presented to the mind two objections—an obstacle in advance of the capillaries, and an interruption to the supply from behind." P. 203.

As the following experiment is conceived by the writer to be free from all such difficulties, we give it in his own words:—

"It is not my intention to examine the phenomena of fœtal circulation, but to allude only to one striking peculiarity, viz. the circulation of the blood in the umbilical vein. This fluid is transmitted from the placenta to the fœtus without the aid of any propulsive organ. The capillaries are, indeed, the only source of motive power shown to exist, and hence, the placenta, separated from the uterus, appeared capable of determining the influence of capillaries, in urging the blood through the long capacious vein. To test the fact, a placenta was procured, twenty minutes after separation from the uterus, and placed with the exception of the cord, in a bladder, which was immersed in water, at the temperature of 100° Fahrenheit. The free extremity of the cord, at the same moment, was elevated to an angle of 30°, resting on the edge of a glass, and at the distance of a foot from the placenta. At the commencement no blood escaped from the vein, but in two minutes from the immersion, it began to flow, and continued for about twenty minutes, and at this time, the glass had received above an ounce.

"Here, then, is an experiment, unexceptionable in its character, demonstrating the power of the capillaries to carry on the circulation, not only in their complicated net-work of vessels, but in larger vessels, ultimately terminating in a capacious vein." P. 205.

In the correctness of the assertion that the experiment is "unexceptionable" we cannot concur; indeed, the author himself observes of it, that "the disengagement of it (the placenta) from the uterus disturbs the normal relations of these vessels," that is the placental arteries, veins and capillaries. It is rather surprising that Dr. Holland, who urges his "objections" somewhat unsparingly, should not have been more judicious in the selection of his own experiment, and particularly that he should not have guarded against "an interruption to the supply from behind." The oozing from the umbilical vein, for, as only an ounce was collected in twenty minutes, the escape of blood could have amounted to nothing more, it may reasonably be inferred depended upon that gradual contraction of the arteries, which it has been distinctly shown by Hunter and many subsequent observers, those vessels possess, by which they are enabled to adapt themselves to the varying bulk of their contents. The great point which the author wishes to establish by the experiment we have detailed, and by his elaborate critiques, is that the capillary vessels, which he states, but erroneously, to be the seat of all the vital actions, assist by their contractility in the circulation.

Without dwelling longer upon this questio vexata, we will only state it is our conviction, that although the capillaries have distinct walls (we have seen them plainly with the microscope, as Schwann and Henle had before, in vessels taken from the pia mater and elsewhere), and although they possess a power of contraction, and especially a capability of becoming preternaturally dilated, yet that they do not aid in the normal circulation of the blood by any propulsive action.

Our limits will not allow us to follow the author in his criticisms relating to the circulation of the acardiac fœtus, which in themselves offer little that is novel. But we must remark in conclusion that, the ingenious experiment of Dr. M. Hall, in which, by placing a heavy probe upon the dorsal fin of the eel, he rendered the circulation in the capillaries *pulsatile,* and thus demonstrated that the single ventricle of the fish does operate through *two sets of capillary vessels,* the branchial and systemic, is an all-important element in this and similar inquiries, and is not liable to be set aside, notwithstanding Dr. Holland's "Objections."

